# Simultaneous integrated boost for adjuvant treatment of breast cancer- intensity modulated vs. conventional radiotherapy: The IMRT-MC2 trial

**DOI:** 10.1186/1471-2407-11-249

**Published:** 2011-06-15

**Authors:** Vasileios Askoxylakis, Alexandra D Jensen, Matthias F Häfner, Leonie Fetzner, Florian Sterzing, Joerg Heil, Christof Sohn, Johannes Hüsing, Uta Tiefenbacher, Frederik Wenz, Jürgen Debus, Holger Hof

**Affiliations:** 1 Department of Radiation Oncology, University of Heidelberg, INF 400, 69120, Heidelberg, Germany; 2 University Breast Center, Department of Gynecology, University of Heidelberg, Vossstr. 9, 69115 Heidelberg, Germany; 3 Coordination Centre for Clinical Trials, University of Heidelberg, Vossstr. 2, 69115 Heidelberg, Germany; 4 Department of Radiation Oncology, University Medical Center Mannheim, Heidelberg University, Theodor-Kutzer-Ufer 1-3, 68135 Mannheim, Germany

## Abstract

**Background:**

Radiation therapy is an essential modality in the treatment of breast cancer. Addition of radiotherapy to surgery has significantly increased local control and survival rates of the disease. However, radiotherapy is also associated with side effects, such as tissue fibrosis or enhanced vascular morbidity. Modern radiotherapy strategies, such as intensity modulated radiotherapy (IMRT), can shorten the overall treatment time by integration of the additional tumor bed boost significantly. To what extent this might be possible without impairing treatment outcome and cosmetic results remains to be clarified.

**Methods/Design:**

The IMRT-MC2 study is a prospective, two armed, multicenter, randomized phase-III-trial comparing intensity modulated radiotherapy with integrated boost to conventional radiotherapy with consecutive boost in patients with breast cancer after breast conserving surgery. 502 patients will be recruited and randomized into two arms: patients in arm A will receive IMRT in 28 fractions delivering 50.4 Gy to the breast and 64.4 Gy to the tumor bed by integrated boost, while patients in arm B will receive conventional radiotherapy of the breast in 28 fractions to a dose of 50.4 Gy and consecutive boost in 8 fractions to a total dose of 66.4 Gy.

**Discussion:**

Primary objectives of the study are the evaluation of the cosmetic results 6 weeks and 2 years post treatment and the 2- and 5-year local recurrence rates for the two different radiotherapy strategies. Secondary objectives are long term overall survival, disease free survival and quality of life.

**Trial Registration:**

ClinicalTrials.gov Protocol ID: NCT01322854.

## Background

Breast cancer is the most common cancer entity in women in developed countries, representing a major health care problem. The disease is diagnosed in about 1.2 million patients and accounts for about 500,000 deaths yearly worldwide [1]. Treatment is based on stage, histology and biomarkers and is commonly multimodal. The development of diagnostic strategies has led through the years to an earlier diagnosis of breast cancer, which resulted in the evolution from entirely surgical treatment into more conservative approaches, replacing mastectomy by breast-conserving surgery followed by radiation therapy in early-stage disease [2]. Adjuvant whole-breast external beam radiotherapy has been shown to result in a significant increase of local control, as well as a significant reduction in the risk of death [3], making the outcome of breast-conserving surgery comparable to mastectomy. Furthermore, additional boost-irradiation to the tumor bed is found to further improve local tumor control. In particular, 10-year cumulative incidence of local relapse in the randomized prospective trial comparing whole-breast irradiation versus whole-breast irradiation and subsequent boost was 10.2% versus 6.2% [4]. Further analyses have revealed an increased benefit for patients younger than 50 years and patients with high grade invasive ductal carcinoma [5].

The most common adjuvant radiotherapy strategy after breast-conserving surgery consists of irradiation of the whole breast using two tangential photon beams during a 5-6 week period, with doses of 1.8-2.0 Gy per fraction to a total dose of approximately 50 Gy. Thereafter, boost irradiation is carried out using electrons and/or photons up to a total dose of 66 Gy, resulting in total treatment duration of 7-8 weeks.

Dose escalation by a sequential boost regimen results in a longer treatment duration. Furthermore, various studies have demonstrated that higher radiation dose is associated with a limited but statistically significant worsening of the cosmetic result, mainly due to breast fibrosis. In particular, the risk of moderate or severe fibrosis at 10 years after breast conserving surgery followed by whole breast irradiation with or without boost-irradiation of 16 Gy was significantly increased for the group of patients that had received a boost (12.6% versus 26.9%) [6].

Another important aspect in radiation therapy of breast cancer is the fact that although radiotherapy is associated with reduced disease-specific mortality, an increase of cardiovascular mortality after radiation treatment has been shown, with a trend towards higher risk for women with left-sided disease [7]. Radiation associated cardiac disease can manifest either as acute injury, i.e. pericarditis, or as late injury, such as congestive heart failure, ischemia, coronary artery disease or myocardial infarction months to years post radiation therapy [8]. Volume-dependent perfusion defects after irradiation of left-sided breast cancer using tangential photon beams to a dose of 50 Gy and electron boost of 16-18 Gy has been revealed for up to 40% of patients within 2 years after treatment [9]. The risk for heart disease post-irradiation is higher for younger patients and is further enhanced by the established use of anthracyclines in multimodal treatment regimes [10].

The limitations in radiation therapy of breast cancer reveal the necessity for the use of advanced radiation therapy strategies that allow a more accurate delivery of radiation dose to the target volume sparing the surrounding healthy tissues. A promising technology to achieve this goal is intensity modulated radiotherapy (IMRT). IMRT is an advanced technique using computer-assisted inverse planning and administering radiation via multiple individual segments. Varying intensities across each beam allow improvement of dose homogeneity. Delivery of irradiation to irregularly shaped targets is optimized with IMRT, while the technology offers the ability to produce concavities in the treatment volume and therefore increase conformality. Trials in breast cancer patients have demonstrated better dose uniformity throughout the breast for intensity modulated radiotherapy with a median of 0.1% of the treatment volume receiving ≥110% of the prescribed dose versus 10% with conventional wedges [11]. Helical tomotherapy is an advanced technology for intensity modulated radiotherapy using an image-guided, dynamic, therapy delivery system, which offers the potential for pretreatment megavoltage CT imaging. Pretreatment imaging for patient positioning verification is of high importance, since it allows corrections for inter-fraction positioning errors, which is a prerequisite for accurate delivery of complex IMRT treatment plans [12]. Studies in breast cancer patients comparing tomotherapy with 3D-planning demonstrated a minimal dose increase but improved dose homogeneity and conformity to the planning target volume [13].

A major advantage of IMRT is also the fact that, in contrast to conventional techniques, it provides the possibility to integrate the boost concept in the daily radiation sessions by increasing the dose per fraction within the boost volume [14]. Hence, overall treatment duration can be shortened by more than one week compared to conventional radiation treatment in patients, who are likely to benefit from an additional boost to the tumor bed.

Thus, aim of this prospective, two-armed, multicenter, randomized trial is to compare the results of intensity-modulated radiotherapy versus conventional radiotherapy for adjuvant treatment of breast cancer. Rationale of the project is the reduction of treatment duration in view of at least consistent radiation toxicity, local control, disease-free survival and overall survival, with emphasis given to the cosmetic results and local control after adjuvant treatment.

## Methods/Design

### Trial organization

The trial has been designed by the study initiators at the Department of Radiation Oncology and the Department of Gynecology of the University of Heidelberg. The trial is carried out at the University of Heidelberg, Department of Radiation Oncology and Department of Radiation Oncology, University Medical Center Mannheim. The University of Heidelberg is responsible for trial management and coordination, as well as quality assurance including reporting, monitoring and database management.

### Study design

**Figure 1:**
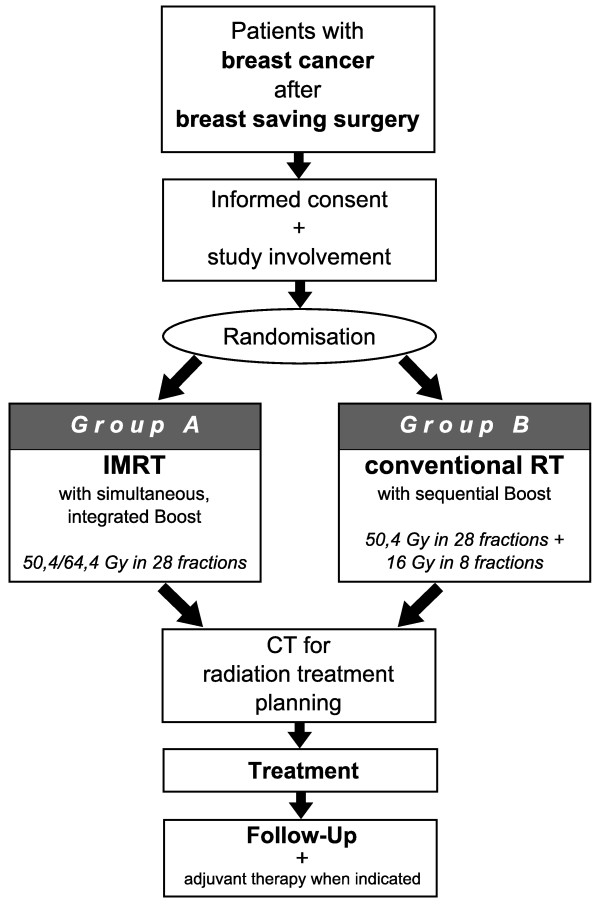
**Trial flow-chart**.

IMRT-MC2 is a prospective, two-armed, multicenter, randomized, phase III trial. The trial workflow and treatment arms are depicted in Figure 1.

Patients fulfilling the inclusion criteria will be randomized into two arms:

#### Arm A- Experimental Arm

IMRT of the whole residual breast to a total dose of 50.4 Gy and 1.8 Gy per fraction with 2.3 Gy per fraction within integrated boost to the tumor bed to a total dose of 64.4 Gy in 28 fractions.

#### Arm B- Conventional Arm

Conventional radiotherapy of the whole residual breast to a total dose of 50.4 Gy and 1.8 Gy per fraction in 28 fractions, followed by consecutive boost to a total dose of 66.4 Gy and 2 Gy dose per fraction in 8 fractions. A total of 36 fractions is given to patients included in arm B.

### Study objectives

Primary objectives of the study are cosmetic result and local control rate after radiotherapy. The cosmetic result will be evaluated at 6 weeks and 2 years post radiotherapy. Local control will be evaluated at 2 and 5 years post treatment.

Secondary objectives of the trial are quality of life at 6 weeks and 2 years after radiotherapy, as well as overall survival and disease free survival at 6 weeks, 2, 5, 10 and 15 years post radiation treatment.

### Investigators

Patient treatment will be performed by radiation oncologists at the Department of Radiation Oncology at the University of Heidelberg and the Department of Radiation Oncology at the University Medical Center Mannheim.

### Data Handling, Storage and Archiving

All findings including clinical and laboratory data will be documented by the investigator or an authorized member of the study team in the subject's medical record and eCRF (electronic Case Report Form). The investigator is responsible for ensuring that all sections of the eCRF are completed correctly and that entries can be verified against source data. In some cases, the eCRF, or part of the eCRF, may also serve as source documents: Karnofsky Performance Status, Documentation of Cosmetic Result and Clinical Examination.

Data will be collected and entered in a study specific database by the Study Center of the Department of Radiation Oncology at the University of Heidelberg and the Study Center of the Department of Radiation Oncology at the University Medical Center Mannheim.

All missing data or inconsistencies will be reported back to the investigators and clarified by the responsible investigator. If no further corrections are to be made in the database it will be declared closed and used for statistical analysis.

The data will be stored and archived according to §13 of the German GCP-Regulation and §28c of the German X-Ray Regulation (RöV) and §87 of the German Radiation Protection Regulation (StrlSchV) for at least 30 years after the trial termination.

Data management for this study will be coordinated by the Coordination Centre for Clinical Trials (KKS) Heidelberg.

### Ethics, informed consent and safety

The final protocol was approved by the University of Heidelberg ethics committee (S-041/2009) and the Federal Office of Radiation Protection (BfS) (Z5-22461/2-2009-018). The trial is sponsored by the German Aerospace Center (DLR)/Federal Ministry of Education and Research (BMBF) of Germany (01ZP0504). This study complies with the Helsinki Declaration and its recent German version, the Medical Association code of conduct, the principles of Good Clinical Practice (GCP) and the Federal Data Protection Act. The trial will be carried out in keeping with local legal and regulatory requirements. The medical secrecy and the Federal Data Protection Act will be followed. The ClinicalTrials.gov Protocol ID is NCT01322854.

### Patient selection

#### Inclusion criteria

Patients meeting the following criteria will be considered for admission to the trial:

• All patients aged >18 years and <70 years after breast conserving surgery.

• Patients aged ≥70 years with following risk factors:

○ Tumor stage ≥T2

○ Multifocal disease

○ Lymphangiosis

○ Extended intraductal component

○ Resection margin ≤3 mm

### Exclusion criteria

Patients presenting with one of the following criteria will not be included in the trial:

• Refusal of the patients to be included in the study

• Karnofsky Performance Score ≤70%

• Metastatic disease (M1)

• Other malignancies

• Previous radiotherapy of the breast

• Pregnancy

### Study plan

502 patients (251 patients in each arm) with breast cancer and previous breast conserving surgery are included in the study according to the criteria above. Eligible patients are informed about participation in the trial with possible benefits and risks, and written informed consent is obtained. Patients are randomized into the two treatment arms after baseline photo-documentation and evaluation of the postoperative cosmetic result through an expert panel and the patient, as well as evaluation of life quality using an EORTC questionnaire.

### Treatment planning and radiation therapy

#### Arm A

After reliable patient positioning, a thoracic CT scan with a slice distance of 3 mm is carried out. Based on the CT data set, radiation treatment planning is performed as inverse planning using the planning tool KonRad^® ^(Siemens OCS, Erlangen, Germany). Multiple fields arranged in a star-shape around the ipsilateral hemithorax are chosen. Intensity modulation is optimized according to the specified dose directions applying an inverse treatment planning technique. 6 MV-photons are used for irradiation. IMRT can be applied either in step-and-shoot technique at a linear accelerator or at a dedicated tomotherapy unit. During treatment, regular image guidance via MV-CT is carried out for positioning control.

The clinical target volume (CTV) for the residual breast tissue and - in certain cases - the locoregional lymphatics, will be defined according to the S3 guidelines of the German Cancer Society [15]. The CTV for Arm A includes the residual breast up to 5 mm under the skin surface. The primary target volume (PTV) includes the CTV with a margin of 10 mm in all directions when optimization via virtual bolus concept is possible. In Arm A/Tomotherapy or Arm A/IMRT without virtual bolus concept the PTV will not be extended over the skin surface. The GTV includes the tumor bed, as defined by preoperative mammography.

IMRT treatment is performed in 28 fractions with 1.8 Gy per fraction to a total dose of 50.4 Gy and integrated boost with 2.3 Gy per fraction to a total dose of 64.4 Gy. This is equivalent to the dose of the conventional treatment arm in consideration of the linear-quadratic model. CTV will be covered by 45 Gy, GTV will be covered by 95% of the prescribed dose i.e. 60.8 Gy. With regard to the organs at risk, less than 10% of the heart volume may receive >30 Gy, while less than 20% of the ipsilateral lung may receive more than 20 Gy. The mean dose to the contralateral breast should be limited to less than 5 Gy.

In case dose to the target volume and dose limits to the organs at risk can not be met at the same time, compliance with the threshold dose in the target volume is paramount. However, dose limitations in contralateral structures have to be met.

Treatment duration is 5^3/5 ^weeks with 5 fractions per week.

#### Arm B

Patient positioning can be performed according to the standards of the participating medical centers and will be controlled weekly by conventional verification films. For treatment in Arm B thoracic scans with a slice distance of 10 mm are allowed.

The clinical target volume (CTV) includes the residual breast to the skin surface and in certain cases the locoregional lymphatics, according to the S3 guidelines of the German Cancer Society [15]. The primary target volume (PTV) includes the CTV with a medio-lateral and cranio-caudal margin of 10 mm, as well as a ventral margin of 20 mm. The GTV includes the tumor bed, as defined by preoperative mammography.

Conventional radiation treatment of the residual breast is performed in 28 fractions with 1.8 Gy per fraction to a total dose of 50.4 Gy. Subsequently, a consecutive boost to the tumor bed is carried out with 2 Gy per fraction in 8 fractions to a total dose of 66.4 Gy. Patients treated in Arm B receive a total of 36 fractions.

CTV will be covered by 45 Gy, GTV will be covered by 95% of the prescribed dose, i.e. 63.1 Gy. In regard to the organs at risk, less than 10% of the heart volume may receive >30 Gy, less than 40% of the ipsilateral lung may receive >20 Gy, while the mean dose in the contralateral breast should be less than 5 Gy.

In case dose to the target volume and dose limits to the organs at risk can not be met at the same time, compliance with the threshold dose in the target volume is paramount. However, dose limitations in contralateral structures have to be met.

Treatment duration is 7^1/5 ^weeks with 5 fractions per week.

### Irradiation of the lymphatics

In both arms irradiation of the lymphatics will be performed when indicated, according to the S3 guidelines of the German Cancer Society [16].

Irradiation of the axillary lymph nodes is recommended in following cases:

• Presence of residual tumor in the axilla

• Clinically apparent tumor spread to the axilla or in case of positive sentinel node biopsy without or after incomplete axillary dissection

Irradiation of the supra-/infraclavicular lymph nodes is recommended in following cases:

• When more than 3 lymph node are positive

• In case of tumor spread to level III

### Follow up

The first radio-oncologic follow-up is planned 6 weeks after treatment completion. Further trial related follow-up visits are scheduled at 2 years and 5 years post treatment. In the time between 6 weeks and 2 years, as well as between 2 and 5 years post treatment, follow-up examinations are conducted by the attending gynecologists, according to the guidelines of the German Cancer Society.

Evaluation of the cosmetic result is conducted 6 weeks and 2 years post treatment. For complete evaluation of overall survival, disease free survival, as well as possible late side-effects, e.g. secondary cancer, a follow-up period of 15 years is necessary. Therefore, further trial related documentation will be performed at 10 and 15 years post treatment.

**Figure 2:**
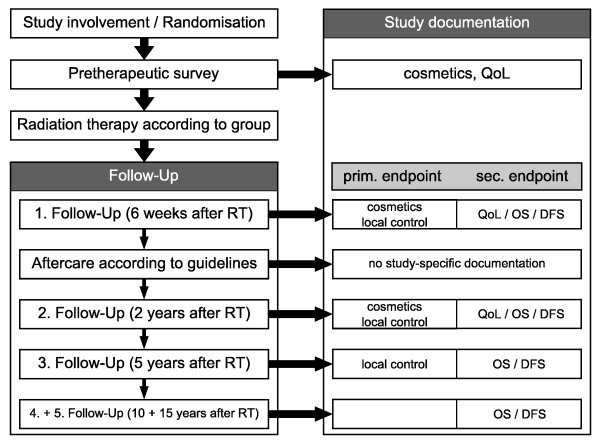
**Trial follow-up**.

An overview of trial related follow-up is shown in Figure 2.

### Evaluation

The cosmetic result is evaluated via photo-documentation. Before radiotherapy, 6 weeks and 2 years post treatment photographs of the patient's torso are taken in a standardized way: 2 frontal photographs from neck to midabdomen (one with arms raised and one with both arms alongside the body) and a profile photograph of the treated breast (arms raised). The pictures are evaluated by two independent investigators. Parameters to be evaluated are skin color, teleangiectasy, scars, shrinking and asymmetry. Evaluation is also carried using a quantitative digitizer scoring system, as described by Vrieling et al. [17]. Four positions are scanned on a frontal view patient photo: the mark at suprasternal notch, the mark at the midline 25 cm inferior to the suprasternal notch, the nipple position of the treated breast and the nipple position of the untreated test. These are used for the calculation of a breast retraction assessment (BRA) score. Furthermore, cosmetic evaluation is based on the standards set forth by the Harvard criteria [18]. An excellent score is given when the treated breast looks essentially the same as the contralateral, untreated breast. A good score is given for minimal but identifiable radiation effects on the treated breast, while a fair score is assigned for significant radiation effects. A poor score is used for severe sequelae of the treated breast.

Tumor manifestations within the residual breast, the regional lymphatics (axillary, parasternal, infra-/supraclavicular) or the radiation field borders are defined as locoregional relapse. Other types of tumor recurrence are considered distant metastases.

Secondary cancers must be confirmed by histological analysis. The results of the analysis should not be consistent to the original histology of the primary tumor. Date and way of diagnosis, as well as the applied dose at the site of origin of the second malignoma are documented.

Quality of life is assessed by means of the EORTC questionnaire QLQ-C30 and the breast cancer questionnaire QLQ-BR23 at 6 weeks and 2 years post treatment. The EORTC QLQ-C30 is an established instrument for measuring quality of life in cancer patients and contains 30 items that measure five functional scales, global quality of life and several cancer related symptoms. The QLQ-BR23 questionnaire is a specific tool, which contains 23 items measuring functioning and symptoms related to breast cancer [19]. Life quality assessment should include: pain/dysaestesia (yes/no), frequence/duration (no/intermittent/permanent) and treatment necessity (no/intermittent/permanent).

### Statistical analysis

#### Primary criteria and hypotheses

Primary aim of the study is the assessment of the cosmetic outcome and local control at 2 years after breast radiotherapy. The question to be investigated is whether intensity modulated radiotherapy (IMRT) is, in spite of the reduced treatment duration, at least equivalent to conventional therapy. The combined null hypothesis (H_0_) is "Conventional therapy is better by more than a pre-specified margin than IMRT with respect to the cosmetic outcome or local control". The alternative hypothesis (H_1_) is: "IMRT is better, or worse at most by the pre-specified margin than conventional radiotherapy with respect to both cosmetic outcome and local control". The null hypothesis will be rejected, if the lower limits of both 95 per cent confidence intervals for the effect of IMRT vs. standard are higher than the respective non-inferiority margin. The non-inferiority margins are set to a relative risk of 4.67 for local control and 1.54 for the BRA measurement of cosmesis. The non-inferiority margin for local control seems very high but it is expected to correspond to an absolute risk difference of 7 percent (from 98 to 91 per cent). The adoption of local control as a primary endpoint despite low probability of loss of local control honours the importance of the endpoint and safeguards against too severe concessions in this respect. As the total radiation dose is equivalent for both arms though, it is expected that there will be no difference in local control.

#### Sample size calculation

The required patient number was assessed using the software R with a simulation of 9999 iterations. 251 patients per trial arm are required for a power of 97.5% with reference to cosmetic result and 82.5% with reference to local control, considering a drop-out rate of 10%. The assumed drop-out rate includes patients, who withdraw their consent to participate after randomization, as well as patients who are not treated according to the protocol, do not meet inclusion criteria or meet exclusion criteria, whose data files are incomplete or whose status is not properly assessed by follow-up examinations.

#### Analysis methods

Differences in the primary endpoint "cosmetic outcome" will be assessed by taking the variable as response in a linear model with the randomized treatment strategy, the age of the patients and the cosmetic parameter at baseline as explanatory variables. Local control will be estimated as the parameter for randomized treatment in a Cox proportional hazard model with age as additional explanatory variable. Approved methods for censored data, such as Kaplan-Meier estimation and/or log-rank test will be applied for statistical analysis of all secondary events.

## Discussion

The role of intensity modulated radiation therapy (IMRT) in the treatment of breast cancer has been subject of investigation in various trials within recent years. Most of the studies have performed dosimetric analyses of IMRT compared to other radiation therapy techniques. One of the first trials to directly compare IMRT tangential photon fields versus tangential photon fields with oblique electron-photon fields with manually optimized beam wedges and wide split tangential photon fields with computer optimized wedge angles in the treatment of breast cancer was performed by Cho et al. [20]. In this study, the root mean square deviation of the differential dose-volume histogram (RMS dDVH), which is a measure of dose homogeneity was found to be lower for IMRT compared to the other techniques, revealing an improved dose coverage to the treatment volume. In the same study, the normal tissue complication probabilities (NTCPs) for the organs at risk, i.e. heart and lungs, were calculated for comparison. The average NTCP for excess late cardiac mortality and radiation pneumonitis was calculated to be lower for IMRT, while the intensity-modulated technique also showed the lowest partial body mean dose. A further study from the same group has shown a 50% reduction of NTCP for late cardiac toxicity using tangential IMRT, compared to conformal tangential fields [21].

The dosimetric advantages of inversely planned IMRT in the treatment of breast cancer have been demonstrated by Thilmann et al. in a plan comparison study including 20 patients [22]. In all cases the homogeneity in the target volume was improved for the inversely planned IMRT compared to the 3D-planned conventional radiotherapy (CRT). The volume of the ipsilateral lung irradiated with a dose higher than 20 Gy was strongly reduced with IMRT compared to CRT (13.1% versus 24.6% respectively). A similar trend was noticed for the heart volume receiving a dose higher than 30 Gy (0.2% for IMRT versus 6.2% for CRT).

More recent studies have also confirmed superior dose distribution and homogeneity for IMRT, while also revealing that the use of intensity modulation leads to a reduction of the dose to the contralateral breast compared to conventional tangential field techniques. Bhatnagar et al. showed a reduction of 36% and 57% at 4 and 8 cm respectively from the center of the medial border of the tangential field on the contralateral breast when IMRT was used compared to 3D-technique, a result which was found to be highly significant [23].

Improvement of dose homogeneity with IMRT in breast cancer has a positive influence on clinical benefits for the patients in terms of reduced acute or late toxicity. Toxicity analysis of 172 breast cancer patients treated with either IMRT or with wedge-based radiotherapy showed a significant reduction in acute dermatitis grade 2 or worse, edema or hyperpigmentation when IMRT was applied. Chronic edema grade 2 or worse was also significantly reduced in favor of IMRT [24]. A significant reduction in the occurrence of acute radiation dermatitis using IMRT has also been demonstrated in a multicenter double-blind, randomized clinical trial of about 330 breast cancer patients, who were treated with either IMRT or standard radiotherapy with wedge compensation to a total dose of 50 Gy in 25 fractions with an additional boost of 16 Gy when necessary [25]. This study demonstrated a significant decrease in the occurrence of moist desquamation during or up to 6 weeks post radiation treatment for the IMRT group. In particular, 31.2% of the patients treated with IMRT showed acute radiation dermatitis, while the value was 47.8% for the standard treatment arm. The same study showed no correlation of IMRT with pain or quality of life, still the presence of moist desquamation did significantly correlate with both pain and a reduced quality of life.

Reduced acute and late toxicity of breast IMRT results in improved cosmetic results. A randomized trial of standard 2D-radiotherapy versus IMRT in about 240 breast cancer patients investigated change in breast appearance scored from serial photographs before and after radiation treatment. This study showed that 58% of the patients treated with 2D-radiotherapy had changes in breast appearance, compared to 40% of the patients treated with IMRT, while significantly fewer patients in the IMRT group had developed palpable indurations of the breast [26].

Several publications have confirmed the feasibility and dosimetric superiority to conventional plans, as well as a decrease of acute and late toxicity compared to conventional radiation therapy in recent year. Despite the fact that breast IMRT is a field of high interest, long-term, clinical data are mostly based on retrospective analyses. One analysis performed by McDonald et al. showed no statistically significant differences in overall survival, disease specific survival, freedom from ipsilateral breast tumor recurrence, distant metastasis, late toxicity or second malignancies between 121 patients treated with IMRT and 124 patients treated with conventional radiotherapy to a median dose of 50 Gy, followed by a boost to a median total dose of 60 Gy [27]. Still, the study showed a slight trend in favor of IMRT with the 7-year freedom from ipsilateral breast tumor recurrence value found to be 95% for IMRT and 90% for conventional radiotherapy.

Therefore, the aim of our prospective, randomized, multicentric, phase III trial is a thorough evaluation of adjuvant IMRT for cosmesis, long-term clinical outcomes and quality of life for breast cancer patients in comparison to conventional 3D radiation therapy. Treatment dose for both arms is calculated to be equivalent in consideration of the linear-quadratic model. Based on the results of previous studies we investigate the hypothesis of a better cosmetic result for breast IMRT with at least consistent radiation toxicity, local control, disease-free survival and overall survival. Furthermore, through boost integration in the IMRT plan, we aim to shorten the therapy duration of about one to two weeks compared to conventional treatment strategies.

## Competing interests

The authors declare that they have no competing interests.

## Authors' contributions

HH, UT, ADJ, JD, CS, FW planned the study. VA, MFH, LF, HH and UT are responsible for patient recruitment. VA, MFH, LF, FS, UT and HH perform planning and radiation therapy. JHu performs biometric and statistical analysis. Medical care and follow up is provided by VA, MFH, LF, JH, UT and HH. VA, JH and HH drafted the manuscript. CS, FW and JD revised the manuscript critically for important intellectual content. All authors have read and approved the final manuscript.

## Pre-publication history

The pre-publication history for this paper can be accessed here:

http://www.biomedcentral.com/1471-2407/11/249/prepub
